# A new species and three new combinations in the genus *Nannengaella* (Physaraceae, Myxomycetes)

**DOI:** 10.3389/fmicb.2026.1733966

**Published:** 2026-05-12

**Authors:** Xuefei Li, Jiajia Wang, Frederick Leo Sossah, Jingyu Wang, Bo Zhang, Xiao Li, Yu Li

**Affiliations:** 1 College of Mycology, Jilin Agricultural University, Changchun, China; 2 Engineering Research Center of Chinese Ministry of Education for Edible and Medicinal Fungi, Jilin Agricultural University, Changchun, China; 3 Industrial Development Institute for Plants, Animals and Fungi Integration of Biyang County, Biyang, China; 4 Council for Scientific and Industrial Research (CSIR), Oil Palm Research Institute, Coconut Research Programme, Sekondi, Ghana; 5 Jilin Academy of Agricultural Sciences, Changchun, China

**Keywords:** multilocus phylogeny, Myxomycetes, *Nannengaella*, new combinations, new species, taxonomy

## Abstract

Myxomycetes (true slime molds) are amoebozoan protists involved in decomposition and nutrient cycling in terrestrial ecosystems. Recent molecular studies have led to major taxonomic revisions in Physaraceae, including the establishment of the genus *Nannengaella* for highly calcified species previously placed in *Physarum*. To refine the taxonomy and distribution of *Nannengaella* in China, we used an integrative approach combining multilocus phylogenetics and morphological examination. A total of 149 specimens from 14 provinces and autonomous regions in China were studied, and phylogenetic analyses were conducted using five loci: nSSU, EF-1α, mtSSU, α-tubulin, and COI. Results confirmed that *Nannengaella* is a distinct and monophyletic lineage within Physaraceae. One new species, *Nannengaella luteotestacea*, is described as a well-supported lineage in the genus, and three former *Physarum* species (*N. herbatica*, *N. cremilutea*, *N. conglomerata*) are recombined into Nannengaella based on molecular and morphological evidence. New distribution records in China are provided for several species: *N. herbatica* from Jilin Province, *N. cremilutea* from Heilongjiang, Shaanxi, and Henan Provinces, and *N. conglomerata*, *N. contexta*, *N. sulphurea*, and *N. leucopus* from Sichuan Province. Detailed morphological descriptions, voucher information, ecological notes, and an identification key are also presented. These findings improve the taxonomic framework of Nannengaella and enhance understanding of its diversity and biogeographic distribution in China.

## Introduction

Myxomycetes (Myxogastria; informally referred to as “slime molds”) are a distinctive lineage of amoebozoan protists. Taxonomically, they are members of the class Myxomycetes (or Myxogastrea) in biological classification, being amoeboid eukaryotes that produce fungus-like sporocarps (Keller et al., 2022) and a monophyletic group within the phylum Amoebozoa of the kingdom Protozoa (Wijayawardene et al., 2020). Their systematic position is also supported by relevant taxonomic and systematic studies (Adl et al., 2012; Cavalier-Smith et al., 2014; Adl et al., 2019; Fiore-Donno et al., 2019). Ecologically, myxomycetes play important roles in terrestrial ecosystems, particularly in the decomposition of organic matter and nutrient recycling, as documented in studies focusing on their biological and ecological characteristics (Stephenson and Stempen, 1994). Beyond their ecological significance, myxomycetes have attracted considerable interest due to their complex life cycles and morphological diversity, which make them valuable models for studying microbial evolution and phenotypic plasticity.

Within this group, the family *Physaraceae* Rostafiński (1873) is characterized by the presence of lime (calcium carbonate) deposits and a wide range of sporocarp morphologies. However, these features exhibit substantial variation and convergence, posing long-standing challenges for taxonomy and phylogenetic inference. The genus *Physarum* Pers. exemplifies this difficulty, having historically been treated as a broad and heterogeneous assemblage encompassing species with considerable morphological variability.

Recent advances in molecular systematics have substantially reshaped our understanding of relationships within *Physaraceae*. Multigene phylogenetic analyses, coupled with detailed morphological reassessment, have demonstrated that *Physarum* in its traditionally circumscription is polyphyletic (García-Martín et al., 2023). As a result, the genus *Nannengaella* was established to accommodate a distinct lineage characterized by highly calcified sporocarps and the presence of a true pseudocolumella (García-Martín et al., 2023). Although this revision has improved generic delimitation, morphological overlap among closely related genera, including *Fuligo*, *Badhamia*, and *Physarum* continues to complicate species-level identification.

The integration of morphological and molecular data has significantly enhanced taxonomic resolution in myxomycetes. Phylogenetic analyses conducted by García-Martín et al. (2023) strongly support the monophyletic status of *Nannengaella*; this conclusion is robustly inferred from sequence data of nSSU, EF-1α, mtSSU, α-tubulin.

Nevertheless, important gaps remain. The global diversity of *Nannengaella* is still incompletely resolved, and cryptic diversity may be underestimated due to limited taxon sampling and reliance on a restricted set of molecular markers. Beyond cryptic diversity, most described *Physarum* species have not yet been sequenced for even a single molecular marker, leaving their phylogenetic positions unclear; some of these species are likely to belong to *Nannengaella*. In addition, ecological traits, life-cycle dynamics, and responses to environmental gradients remain poorly characterized. Ultrastructural features, such as the ontogeny of the capillitium and lime knot formation, are also insufficiently studied, limiting their utility as diagnostic characters.

In this study, we address these gaps by integrating multigene phylogenetic analyses (nSSU, EF-1α, mtSSU, α-tubulin, COI) with detailed morphological observations to refine the taxonomy of *Nannengaella* in China. We describe one new species, propose three new combinations, and provide updated distributional records for the genus. Furthermore, we present comprehensive morphological descriptions, voucher information, ecological data, and an identification key to facilitate accurate species delimitation. These findings contribute to a more robust taxonomic framework for *Nannengaella* and improve our understanding of its diversity, evolution, and biogeographic distribution.

## Materials and methods

### Sampling and specimens

A total of 149 specimens of *Nannengaella* were collected from multiple provinces and regions of China, including Heilongjiang, Henan, Hubei, Gansu, Jiangsu, Jiangxi, Jilin, Liaoning, Shaanxi, Shandong, Sichuan, Yunnan, Guangxi Zhuang Autonomous Region, and the Inner Mongolia Autonomous Region. In addition, previously collected materials preserved in the Herbarium of Mycology, Jilin Agricultural University (HMJAU), were examined. All voucher specimens generated in this study are deposited in HMJAU.

### Morphological study

Macroscopic characteristics, including sporocarp color and texture, stalk morphology, and columella structure, were examined using a Zeiss Axio Zoom V16 dissecting microscope (Carl Zeiss Microscopy GmbH, Göttingen, Germany). Photographs were obtained using a Leica M165 stereomicroscope (Leica Microsystems, Wetzlar, Germany).

For microstructural observations, dried specimens were rehydrated in 3% KOH and examined under a Zeiss Axio Imager A2 light microscope equipped with a Zeiss Axiocam 506 camera. Diagnostic features included spore size and ornamentation, capillitium morphology and pigmentation, and the presence of lime knots. For each specimen, at least 30 mature spores were measured.

Ultrastructural feature of spores and the capillitium were further investigated using a JSM-IT800 scanning electron microscope (JEOL, Tokyo, Japan). Color terminology follows the *Flora of British Fungi Colour Identification Chart* (Royal, 1969). The range of variation in size for sporocarps and spores is described as (minimum–)25% quartile–75% quartile(–maximum), following the latest literature standards (Leontyev et al., 2023).

### DNA extraction, PCR amplification, and sequencing

Genomic DNA was extracted from sporocarps using the TIANamp Micro DNA Kit (Tiangen Biotech Co., Ltd., Beijing, China), following the manufacturer’s instructions. Five loci were targeted for phylogenetic analyses: nuclear small subunit rDNA (nSSU), elongation factor 1-alpha (EF-1α), mitochondrial small subunit rDNA (mtSSU), α-tubulin, and cytochrome c oxidase subunit I (COI). Standard primer pairs were employed: S2(F)/SR4Dark(R) for nSSU (Fiore-Donno et al., 2008, 2012); PB1F/PB1R for EF-1α (Novozhilov et al., 2013); Kmit-F/Kmit-R for mtSSU (Lado et al., 2022); COMF/COMRs for COI (preferred longer fragment) (Liu et al., 2015; Novozhilov et al., 2019), with COIF1/COIR1 used as an alternative (shorter fragment) (Feng and Schnittler, 2015); and KTub-F2/KTub-R1 for α-tubulin (longer fragment) (García-Martín et al., 2023), with KTub-F3/KTub-R1 as a backup (shorter fragment) (García-Martín et al., 2023).

PCR reactions were performed in 25 μL volumes containing 12.5 μL of 2 × EasyTaq^®^ PCR SuperMix (TransGen Biotech Co., Ltd., Beijing, China), 1 μL of each primer (10 μM), 3 μL of DNA template, and 7.5 μL of ddH2O. Amplifications were carried out under locus-specific thermocycling conditions.

For nSSU and mtSSU, the cycling protocol consisted of an initial denaturation at 94°C for 1 min; 30 cycles of 94°C for 1 min, 52°C for 1 min, and 72°C for 3 min; and a final extension at 72°C for 10 min. EF-1α amplification was performed with an initial denaturation at 95°C for 5 min; 36 cycles of 95°C for 30 s, 65.4°C for 30 s, and 72°C for 1 min; and a final extension at 72°C for 10 min. For the COI, two primer pairs were used. The COMF/COMRs pair targeted a longer fragment (95°C for 5 min; 36 cycles of 95°C for 30 s, 52°C for 20 s, and 72°C for 1 min; and a final extension at 72°C for 10 min), while COIF1/COIR1 amplified a shorter fragment under similar conditions with an annealing temperature of 50.7°C. For α-tubulin, KTub-F2/KTub-R1 and KTub-F3/KTub-R1 were used, with annealing temperatures of 52 and 54°C, respectively.

PCR products were verified by electrophoresis on 1% agarose gels stained with ethidium bromide, purified, and sequenced using Sanger sequencing. All newly generated sequences were deposited in GenBank. For each newly described species, sequences were obtained from at least two independent collections (Table 1).

**TABLE 1:** Taxa included in the phylogenetic analyses and their corresponding GenBank accession numbers for the five loci (nSSU, EF-1α, mtSSU, α-tubulin, and COI).

Scientific name	Voucher/specimen numbers	GenBank accession numbers				
		nSSU	*EF-1*α	mtSSU	α *-Tub*	*COI*
*Amaurochaete comata*	AMFD171	AY842031	AY842029	/	/	/
*Didymium dubium*	K7	AM231294	/	/	/	/
*D. dubium*	MA-Fungi 80036	MW240327	MW240059	/	/	/
*D. melanospermum*	MA-Fungi 62790	MG963667	MW240068	/	/	/
*D. melanospermum*	MA-Fungi 91238	MG963668	MG963497	/	/	/
*D. nivicola*	AH19667	MT227019	MT230908	/	/	/
*D. nivicola*	MA-Fungi 90573	MT227090	MT230925	/	/	/
*D. pseudonivicola*	MA-Fungi 90587	MT227099	MT230927	/	/	/
*D. pseudonivicola*	MA-Fungi 90601	MT227112	MT230931	/	/	/
*D. yulii*	HMJAU M3001	MF149870	MK905754	/	/	/
*D. yulii*	HMJAU M3002	MF149871	MK905755	/	/	/
*Enerthenema intermedium*	MM-21635	DQ903688	/	/	/	/
*E. melanospermum*	MM-28388	DQ903689	/	/	/	/
*E. papillatum*	AMFD141	AY643823	/	/	/	/
*Lamproderma aeneum* s. lat.	MA-Fungi 81947	MW240352	MW240092	/	/	/
*L. aeneum* s. lat.	MA-Fungi 86925	MW240353	MW240093	/	/	/
*Lamproderma aeneum* s. lat.	MA-Fungi 90422	MW240354	MW240094	/	/	/
*L. ovoideum*	Sc30802	MN595543	MN596918	/	/	/
*Macbrideola oblonga*	M. Schnittler	DQ903682	/	/	/	/
*Meriderma aggregatum*	AMFD135	DQ903669	/	/	/	/
*M. carestiae*	AMFD173	DQ903671	/	/	/	/
*M. fuscatum*	MM-24907	DQ903668	/	/	/	/
*Nannengaella alpestris*	MA-Fungi 80037	/	/	MW240243	MW239967	/
*N. alpestris*	MA-Fungi-35213	/	MW240114	MW240242	MW239966	/
*N. alpina*	Sc29903	MH930698	MW701648	N/A	/	/
*N. alpina*	Sc29908	MW693002	MW701649	N/A	/	/
*N. alpina*	Sc29955	MW693003	MW701650	N/A	/	/
***N. conglomerata***	**HMJAU M20366-1**	**PP981633**	**PP982068**	**PQ007888**	/	**PP982162**
***N. conglomerata***	**HMJAU M20366-2**	**PP981634**	**PP982069**	**PQ007889**	/	**PP982163**
*N. contexta*	MA-Fungi 73321	MF352474	MF352523	MW240264	/	/
*N. contexta*	MA-Fungi 68752	MF352473	MF352522	/	MW239980	/
***N. contexta***	**HMJAU M20354-1**	/	**PP982066**	**PQ007886**	/	/
***N. contexta***	**HMJAU M20354-2**	/	**PP982067**	**PQ007887**	/	/
***N. cremilutea***	**HMJAU M10217**	**PP981659**	**PP982049**	**PQ007870**	**PP981956**	/
***N. cremilutea***	**HMJAU M20300**	/	**PP982051**	/	/	/
*N. globulifera*	MA-Fungi 46711	MW240379	/	MW240268	MW239990	/
*N. globulifera*	MA-Fungi 51647	MF352479	MF352528	MW240269	MW239991	/
*N. globulifera*	MA-Fungi 51815	/	MW240128	/	MW239992	/
***N. herbatica***	**HMJAU M20342-1**	**PP981629**	**PP982062**	**PQ007882**	/	/
***N. herbatica***	**HMJAU M20342-2**	**PP981630**	**PP982063**	**PQ007883**	/	/
***N. leucopus***	**HMJAU M20301**	**PP981621**	**PP982053**	**PQ007874**	/	/
***N. leucopus***	**HMJAU M20327**	**PP981623**	**PP982055**	**PQ007876**	/	/
*N. mellea*	MA-Fungi 87986	MF352485	MF352535	MG963630	MG963777	/
*N. mellea*	MA-Fungi 60314	/	MG963527	MG963626	MG963773	/
*N. mellea*	MA-Fungi 60322	MW240383	MG963528	MG963628	MG963775	/
*N. mellea*	MA-Fungi 69850	MF352484	MF352534	MG963629	MG963776	/
***N. mellea***	**HMJAU M10127**	**PP981657**	**PP982096**	**PQ007863**	**PP981950**	**PP982149**
***N. mellea***	**HMJAU M10204**	**PP981612**	**PP982037**	/	/	**PP982151**
***N. mellea***	**HMJAU M20133**	/	**PP982098**	/	/	/
***N. mellea***	**HMJAU M20286**	**PP981614**	**PP982039**	**PQ007865**	**PP981952**	**PP982153**
***N. mellea***	**HMJAU M20329**	**PP981616**	**PP982041**	**PQ007867**	**PP981954**	**PP982155**
***N. mellea***	**HMJAU M20346**	**PP981618**	**PP982043**	**PQ007869**	/	**PP982157**
***N. mellea***	**HMJAU M20727**	/	**PP982045**	/	/	/
***N. mellea***	**HMJAU M20728**	/	**PP982046**	/	/	/
***N. mellea***	**HMJAU M20365**	**PP981619**	**PP982047**	/	/	**PP982158**
***N. luteotestacea***	**HMJAU M10278-1**	**PP981653**	**PP982092**	**PQ007857**	/	**PP982145**
***N. luteotestacea***	**HMJAU M10278-2**	**PP981654**	**PP982093**	**PQ007858**	/	**PP982146**
***N. plicata***	**HMJAU M20302**	**PP981610**	**PP982035**	**PQ007859**	**PP981948**	/
***N. plicata***	**HMJAU M20303**	**PP981655**	**PP982094**	**PQ007861**	/	**PP982147**
***N. plicata***	**HMJAU M20348**	**PP981631**	**PP982064**	**PQ007884**	/	**PP982160**
*N. sulphurea*	MA-Fungi 81473	MW240395	MW240145	MW240293	MW240017	/
***N. sulphurea***	**HMJAU M20288**	**PP981661**	/	**PQ007872**	/	/
***N. sulphurea***	**HMJAU M20333**	**PP981625**	**PP982057**	**PQ007878**	/	/
***N. sulphurea***	**HMJAU M20334**	**PP981627**	**PP982059**	**PQ007880**	/	/
***N. sulphurea***	**HMJAU M20341**	/	**PQ441958**	/	/	/
*Physarum atacamense*	MA-Fungi 88415	MG963684	MG963518	MW240248	MG963761	/
*P. atacamense*	MA-Fungi 88445	MW240377	MW240120	MW240249	MW239971	/
*P. biyangense*	HMJAU M20349-1	PP951388	PP948809	PQ007847	/	/
*P. biyangense*	HMJAU M20349-2	PP951389	PP948810	PQ007848	/	/
*P. bogoriense*	MA-Fungi 57191	MF352470	MF352516	MG963610	MG963762	/
*P. bogoriense*	MA-Fungi 69863	MG963685	MG963519	MG963611	MG963763	/
*P. cinereum*	MA-Fungi 63822	/	MF352517	MW240261	/	/
*P. cinereum*	MA-Fungi 70925	/	MF352519	MW240262	/	/
*P. didermoides*	MA-Fungi 51819	/	/	MW240265	MW239987	/
*P. didermoides*	MA-Fungi 71195	MW240378	/	MW240267	/	/
*P. jilinense*	HMJAU M20367-1	PP951401	PP948832	PQ007849	/	/
*P. jilinense*	HMJAU M20367-2	PP951402	PP948833	PQ007850	/	/
*P. licheniforme*	MA-Fungi 73290	MF352481	MF352530	MG963619	MG963768	/
*P. licheniforme*	MA-Fungi 73293	MG963689	MG963524	MG963620	MG963769	/
*P. neoovoideum*	HMJAU M20294-1	PP951386	/	PQ007833	/	/
*P. neoovoideum*	HMJAU M20294-2	PP951387	/	PQ007834	/	/
*P. nigritum*	HMJAU M20276-1	PP951382	PP948805	PQ007813	PP981930	/
*P. nigritum*	HMJAU M20277-1	PP951381	/	PQ007815	/	PP968033
*P. nivale*	MA-Fungi 72831	MF352486	MF352536	MG963633	MG963779	/
*P. nivale*	MA-Fungi 73457	MF352487	MF352537	MG963634	/	/
*P. polygonosporum*	MA-Fungi 90742	MF352465	MF352510	MG963637	MG963781	/
*P. polygonosporum*	MA-Fungi 90752	MW240387	/	MW240279	MW240004	/
*P. pseudonotabile* s. lat.	LE255432	LT670439	KF250465	/	/	/
*P. pseudonotabile* s. lat.	LE255703	LT670568	KF250468	/	/	/
*P. stellatum*	LE297729	MW693019	MW701666	/	/	/
*P. stellatum*	LE297741	MW693020	MW701667	/	/	/
*P. straminipes*	MA-Fungi 70363	MF352489	MF352543	MW240291	MW240015	/
*P. straminipes*	MA-Fungi 87865	MW240394	/	MW240292	MW240016	/
*P. vernum*	Sc30091	MW693021	MW701668	/	/	/
*P. vernum*	Sc30257	MH930744	MW701669	/	/	/
*P. viride*	LE302489	MW693022	MW701670	/	/	/
*P. viride*	LE317322	MW693024	MW701672	/	/	OP616654
*Stemonitis flavogenita*	AMFD2005	AF239229	AY643819	/	/	/
*Symphytocarpus impexus*	/	AY230188	/	/	/	/

Bold fonts indicate the newly generated sequences in this study.

### Phylogenetic analyses

Phylogenetic datasets were constructed by integrating morphological identifications with BLAST-based sequence retrieval across the five loci (nSSU, EF-1α, mtSSU, α-tubulin, COI). The final dataset comprised newly generated sequences, 167 representative sequences of *Nannengaella* retrieved from GenBank, and additional sequences from related genera used as outgroups. Each locus was aligned using MAFFT v7.490 (Katoh and Standley, 2013) with the “—auto” strategy and normal alignment mode, and manually adjusted in BioEdit v7.1.3 (Hall, 1999). Ambiguously aligned regions were inspected and excluded where necessary. Individual alignments were concatenated into a combined dataset using PhyloSuite.

Model selection and partitioned alignment evaluation were implemented using ModelFinder (Kalyaanamoorthy et al., 2017) within IQ-TREE v1.6.12 (Nguyen et al., 2015). Maximum likelihood (ML) analyses were conducted under the best-fit models identified for each partition (TIM2 + F + I + G4 for nSSU, GTR + F + I + G4 for EF-1α/α-tubulin, TPM3u + F + R3 for mtSSU, TVM + F + G4 for COI), with 1,000 ultrafast bootstrap replicates to assess nodal support.

Bayesian inference (BI) was performed via MrBayes integrated in PhyloSuite v1.2.2 (Zhang et al., 2020; Xiang et al., 2023), utilizing the same partition-specific substitution models as ML analyses. Two independent runs with four Markov chains were executed for 2 million generations, with sampling conducted every 1,000 generations. Convergence was verified by effective sample size (ESS) values (> 200 for all parameters) and an average standard deviation of split frequencies converging to 0.0053. The initial 25% of samples were discarded as burn-in, with remaining posterior samples retained for downstream phylogenetic interpretation. Phylogenetic trees were visualized and annotated using iTOL v6.9.

## Results

### Phylogenetic analyses

**FIGURE 1:**
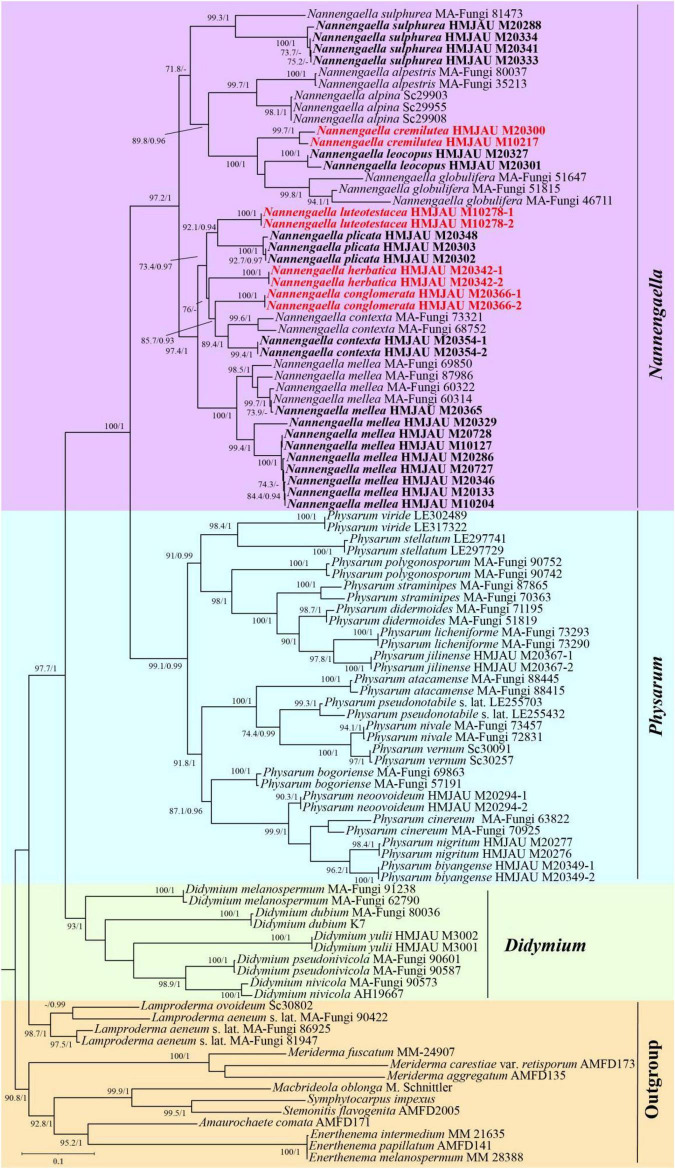
Maximum likelihood phylogenetic tree inferred from the concatenated dataset of five loci (nSSU, EF-1α, mtSSU, α-tubulin, and COI) for *Nannengaella* species. Support valuesat the nodes represent maximum likelihood bootstrap (UBS) percentages and Bayesian posterior probabilities (PP). Taxa newly generated in this study are shown in bold, whereas newly described species and new combinations are highlighted in red.

Phylogenetic relationships were inferred from a concatenated dataset of five loci (nSSU, EF-1α, mtSSU, α-tubulin, and COI) comprising 99 samples representing 45 taxa. The final alignment included 263 sequences, of which 86 were newly generated in this study (21 nSSU, 27 EF-1α, 21 mtSSU, 5 α-tubulin, and 12 COI). The combined dataset consisted of 9,800 aligned characters, including gaps (nSSU: 6,429; EF-1α: 1,139; mtSSU: 436; α-tubulin: 1,049; COI: 747) (Supplementary material). Twelve taxa from the families *Stemonitaceae* and *Lamprodermataceae* were designated as outgroups. Phylogenetic tree comparisons were conducted, and both Maximum Likelihood (ML) and Bayesian Inference (BI) analyses yielded congruent topologies. Consequently, the ML tree (Figure 1) is presented, with support values manually mapped to the corresponding nodes; nodal support is indicated by bootstrap values (BS) and BI posterior probabilities (PP).

The phylogeny resolved two major lineages within *Physaraceae* corresponding to *Nannengaella* and *Physarum* (Figure 1). The *Nannengaella* clade was recovered as sister to *Physarum* with strong support (BS = 100%, PP = 1) (Figure 1). Within *Nannengaella*, a distinct lineage corresponding to *Nannengaella luteotestacea* sp. nov., was recovered as distinct and well supported (BS = 92.1%, PP = 0.94). In addition, three previously described species, *N. conglomerata*, *N. cremilutea*, and *N. herbatica*, were nested within the *Nannengaella* clade, supporting their transfer to this genus. New distributional records were identified for several taxa. *Nannengaella herbatica* was recorded for the first time from Jilin Province, while *N. cremilutea* was newly documented from Heilongjiang, Shaanxi, and Henan Provinces. Furthermore, *N. conglomerata*, *N. contexta*, *N. sulphurea*, and *N. leucopus* were newly recorded from Sichuan Province.

### Taxonomy

***Nannengaella luteotestacea*** X.F. Li, B. Zhang & Y. Li, sp. nov.

MycoBank: MB855045

Figure 2

**FIGURE 2:**
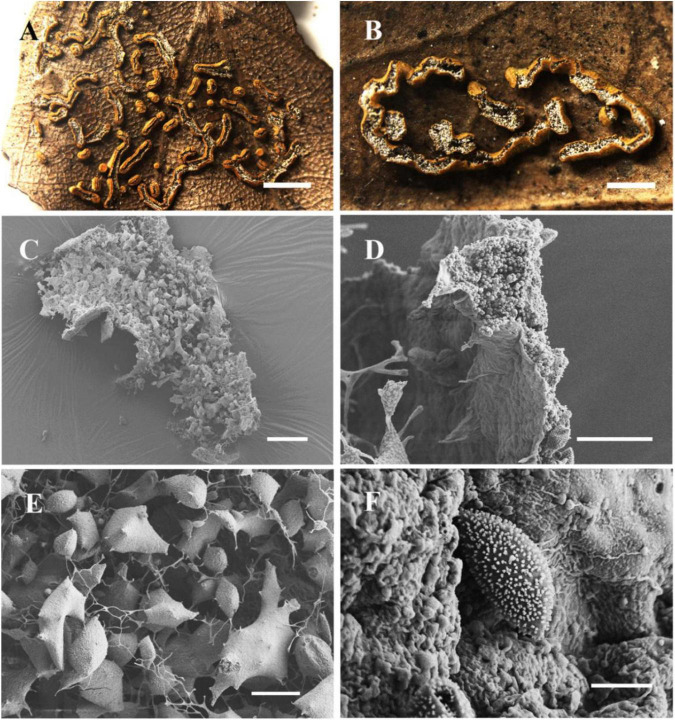
Habitat and microstructure of *Nannengaella luteotestacea* (HMJAU M10278 holotype). **(A,B)** Sporocarps and plasmodiocarps. **(C,E)** Capillitium and lime nodes by SEM. **(D)** Peridium by SEM. **(F)** Spores by SEM. Scale bars: **(A)** = 2 mm; **(B)** = 1 mm; **(C)** = 100 μm; **(D,E)** = 40 μm; **(F)** = 5 μm.

**Etymology:** The epithet “*luteotestacea*” refers to the deep yellow to orange-yellow color of the plasmodiocarps.

**Diagnosis:** Distinguished by dark-yellow, sessile plasmodiocarps that are circular to reticulate in habit, a double-layered peridium, irregular dehiscence lacking a preformed line, absence of surface wrinkles, and spores 8–9 μm in diameter.

**Type:** China, Jiangxi Province, Fuzhou City, Tang Xianzu Memorial Hall, on decaying leaves, 17 June 2013, B. Zhang (HMJAU M10278, holotype).

**Description:** Fructifications mainly plasmodiocarps, curved-linear, circular to reticulate, occasionally forming spherical sporangia, dark-yellow, often fading with age, sessile, readily dehiscence, often persisting as remnants after dehiscence. Columella absent. Peridium thin, double-layered; dark yellow, outer surface covered with calcareous particles, often brown due to lack of calcium at the plasmodiocarp base; the inner layer membranous, colorless, and transparent. Capillitium dense, colorless, with expanded membranous areas. Lime nodes white, yellowish by transmitted light, large, 25–95 × 15–75 μm, circular to polygonal, sometimes forming a central pseudocolumella. Spores dark brown in mass, light brown by transmitted light, globose, (7.5–) 8–9 μm in diam., warted.

**Habitat:** On decaying leaves.

**Distribution in China:** Jiangxi Province.

**Global distribution:** Known only from China.

**Notes:** *Nannengaella luteotestacea* is morphologically and phylogenetically distinct from *P. bogoriense*, *P. hongkongense*, *P. serpula*, and *N. plicata*. In multigene phylogenies, the new species forms a distinct, well-supported lineage. Morphologically, it is characterized by dark-yellow, sessile plasmodiocarps (easily fading, with a brownish calcium-free base), a double-layered peridium (vs. three-layered peridium in *P. bogoriense* and *P. hongkongense*), irregular dehiscence without preformed lines or longitudinal folds (vs. stellate dehiscence with persistent reflexed lobes in *P. bogoriense*, and dehiscence along pre-formed lines in *P. hongkongense*), and abundant, large polygonal lime nodes (25–95 × 15–75 μm) that frequently aggregate to form a pseudocolumella (vs. small, scattered lime nodes in the two *Physarum* species). Sporophores of *N. luteotestacea* are non-compressed, in contrast to the laterally strongly compressed sporophores of *P. hongkongense* and the non-compressed ochre-brown sporophores of *P. bogoriense*; its spores are light brown, (7.8–) 8.0–9.0 μm in diameter, with uniform warts, differing from the violet-brown, clustered-wart spores (7.5–10 μm) of *P. bogoriense* and the smaller light brown, uniformly warted spores (7.5–8 μm) of *P. hongkongense*. Compared with *P. serpula*, the new species differs in having a double-layered peridium (vs. single-layered) and white lime nodes with smaller spores (8–9 μm vs. 10–13 μm). In contrast to *N. plicata*, *N. luteotestacea* lacks longitudinal surface wrinkles, exhibits irregular dehiscence (vs. dehiscence via preformed lines), and has darker yellow plasmodiocarps. The detailed morphological features are compared in Table 2.

**TABLE 2:** Comparison of morphological characteristics between *N. luteotestacea* and its related species.

Characteristic	*N. luteotestacea*	*P. bogoriense*	*P. hongkongense*	*P. serpula*	*N. plicata*
Phylogenetic status	Forms a distinct and well-supported lineage in multigene phylogeny	Known species of the *Physarum*	Known species of the *Physarum*	Known species of the *Physarum*	Known species of the *Nannengaella*
Sporophore morphology	Plasmodiocarps, dark yellow, non-compressed, easily fading, brownish at the calcium-free base	Plasmodiocarps, light reddish brown,reddish brown, non-compressed	Plasmodiocarps, bright yellow, or ochre yellow, strongly laterally compressed, constricted at the base	Plasmodiocarps, orange-yellow	Plasmodiocarps, light yellow or bright yellow
Peridium	Double-layered	Three-layered	Three-layered:	Single-layered	Double-layered
Dehiscence pattern	Irregular dehiscence, without preformed lines or longitudinal folds	Polygonal dehiscence at the upper part, stellate dehiscence at the side with persistent, reflexed lobes	Dehiscence along pre-formed lines	Irregular dehiscence	Dehiscence along pre-formed lines, with longitudinal surface wrinkles
Lime nodes	Abundant, large, polygonal, white, often form a pseudocolumella	Small, rounded to polygonal, white, scattered	Small, rounded to polygonal, white, scattered	Yellowish-white, scattered	Polygonal, white
Spores	Dark brown in mass, light brown under transmitted light	Dark brown in mass, violet-brown under transmitted light	Blackish-brown in mass, light brown under transmitted light	Light brown under transmitted light	Light brown under transmitted light
Spore size	(7.8–)8.0–9.0 μm	7.5–10 μm	7.5–8 μm	10–13 μm	8–9 μm
Spore ornamentation	Verrucose (uniform warts)	Verrucose (warts sometimes clustered)	Verrucose (uniform warts)	Verrucose	Verrucose

***Nannengaella cremilutea*** (Y.F. Chen & C.H. Liu) **X.F. Li, B. Zhang & Y. Li, comb. nov.**

MycoBank: MB855053

Figure 3

**FIGURE 3:**
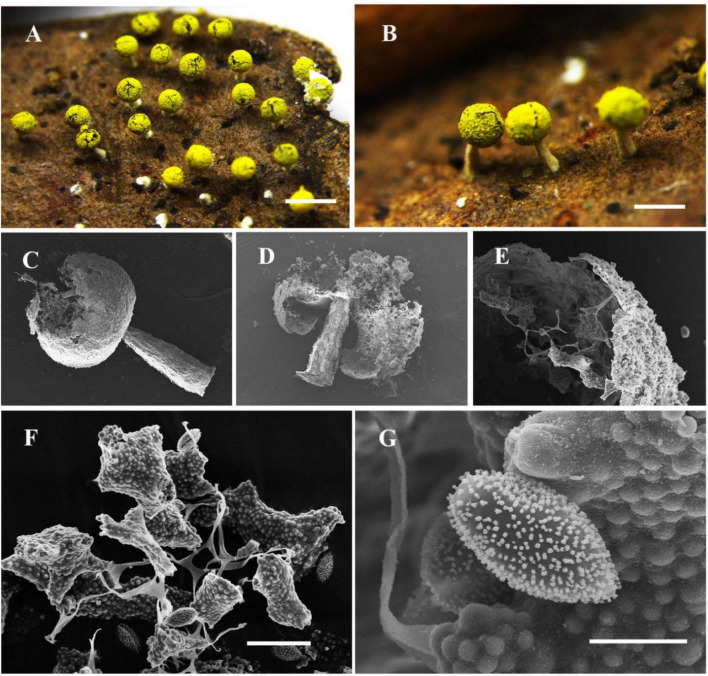
Habitat and microstructure of *Nannengaella cremilutea* (HMJAU M10217). **(A,B)** Sporocarps. **(C,D)** Sporocarp by SEM. **(E)** Peridium by SEM. **(F)** Capillitium and lime nodes by SEM. **(G)** Spores by SEM. Scale bars: **(A)** = 1 mm; **(B)** = 500 μm; **(F)** = 20 μm; **(G)** = 5 μm.

**Basionym:** *Physarum cremiluteum* Y.F. Chen & C.H. Liu, in Liu & Chen, *Taiwania* 43(3):186 (Liu and Chen, 1998).

**Description:** Sporocarps gregarious, stipitate, globose or subglobose, cream-yellow, lemon-yellow or bright yellow-green, 0.4–0.5 mm in diam., and 0.8–0.9 mm in total height; basal part of the sporangium non-calcareous, with a blue or purple iridescence. Stalk white, calcareous, stout, tapering upwards, 0.4–0.5 mm long. Columella absent. Hypothallus small, membranous. Peridium membranous, covered with light yellow lime granules, dehiscing petaloidally. Capillitium netted, with abundant cream-yellow lime nodes that are polygonal to fusiform; connecting threads colorless and transparent. Spores dark in mass, violaceous brown by transmitted light, globose, (7.5–) 8–10 (–11) μm in diam., minutely warted.

**Habitat:** On decaying leaves.

**Distribution in China:** Taiwan Province, Sichuan Province, Hubei Province, Jilin Province, Shaanxi Province, Henan Province, Heilongjiang Province.

**Global distribution:** China and Japan.

**Specimens examined:** China, Jilin Province, Jian City, on decaying leaves, 5 Oct. 2000, Tolgor (HMJAU M20974, HMJAU M20975); China, Jilin Province, Antu County, Erdaobaihe Town, Changbai Mountain Scenic Area, Hunting Ground, on decaying leaves, 22 July 2012, B. Zhang (HMJAU M10162, HMJAU M21012); China, Jilin Province, Changchun City, Jingyuetan national forest park, on decaying leaves, 8 Sept. 2015, B. Zhang (HMJAU M21009); China, Jilin Province, Baishan City, Fusong County, Songjianghe National Forest Park, on decaying leaves, 8 July 2018, B. Zhang (HMJAU M20966); China, Jilin Province, Shulan City, Jiulongshan National Forest Park, on decaying leaves, 29 July 2022, X.F. Li, X.Y. Yang (HMJAU M21028, HMJAU M21029); China, Jilin Province, Jiaohe City, Red Leaf Valley Scenic Area, General’s Altar, on decaying leaves, 31 July 2022, X.F. Li (HMJAU M21013, HMJAU M21014); China, Jilin Province, Tonghua City, Huinan County, Sanjiaolongwan Nature Reserve, on decaying leaves, 10 Aug. 2022, X.F. Li (HMJAU M21011). China, Sichuan Province, Garze Tibetan Autonomous Prefecture, Gexigou National Nature Reserve, on decaying leaves, 3 Aug. 2012, B. Zhang (HMJAU M21049, HMJAU M21050, HMJAU M21051, HMJAU M21052, HMJAU M21053, HMJAU M21054, HMJAU M21055); China, Sichuan Province, Liangshan Yi Autonomous Prefecture, Mianning County, Yihai Scenic Area, on decaying leaves, 6 July 2013, B. Zhang (HMJAU M21026, HMJAU M21027); China, Sichuan Province, Liangshan Yi Autonomous Prefecture, Mianning County, Lingshan Temple, on decaying leaves, 12 Julu 2013, B. Zhang (HMJAU M10177, HMJAU M10200, HMJAU M10198, HMJAU M10168, HMJAU M10159, HMJAU M10150, HMJAU M10210, HMJAU M10190, HMJAU M10195, HMJAU M10128, HMJAU M10207, HMJAU M10182, HMJAU M10152, HMJAU M10122, HMJAU M10216, HMJAU M10137, HMJAU M21030, HMJAU M21031, HMJAU M21032, HMJAU M21033, HMJAU M21034, HMJAU M21035, HMJAU M21036, HMJAU M21037). China, Hubei Province, Shiyan City, Fang County, on decaying leaves, 18 Sept. 2013, B. Zhang (HMJAU M10187). China, Shaanxi Province, Shangluo City, Niubeiliang National Forest Park, on decaying leaves, 21 July 2014, B. Zhang (HMJAU M20971, HMJAU M20972, HMJAU M20973). China, Henan Province, Nanyang City, Neixiang County, Baotianman National Nature Reserve, on decaying leaves. 30 June 2015, B. Zhang (HMJAU M21006, HMJAU M21007, HMJAU M21008). China, Heilongjiang Province, Heihe City, Sunwu County, Shengshan Fortress, on decaying leaves, 13 Aug. 2022, X.F. Li (HMJAU M20969); China, Heilongjiang Province, Heihe City, Sunwu County, Near Yijiazi Mountain, on decaying leaves, 15 Aug. 2022, X.F. Li (HMJAU M20967).

**Notes:** The transfer of this species to *Nannengaella* is supported by its placement within the *Nannengaella* clade in the multigene phylogeny and by its concordant morphology, including calcified structures and typical lime-node development. Morphologically, *N. cremilutea* is distinguished by cream-yellow sporocarps and lime nodes, and by a short, white, calcareous stalk. It resembles *N. mellea*, but the latter has light yellow to dark orange sporocarps and a conical columella, whereas *N. cremilutea* lacks a columella and has uniformly cream-yellow sporocarps. It also differs from *Physarum tenerum*, which lacks the characterstic short calcareous stalk and polygonal lime nodes of *N. cremilutea*. Compared to other congeners, this species is relatively easy to recognize. Due to the uncertainty of environmental changes, after observing a large number of specimens, we found that the spore diameter of this species is slightly larger than the original description.

***Nannengaella herbatica*** (Shuang L. Chen & Yu Li) X.F. Li, B. Zhang **&** Y. Li, comb. nov.

MycoBank: MB855054

Figure 4

**FIGURE 4:**
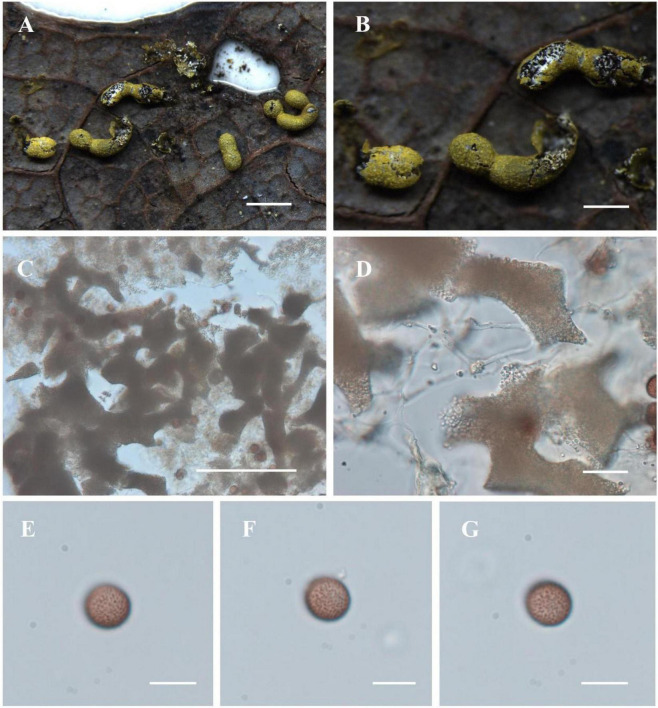
Habitat and microstructure of *Nannengaella herbatica* (HMJAU M20342). **(A,B)** Sporocarps. **(C,D)** Capillitium and lime nodes by TL. **(E–G)** Spores by TL. Scale bars: **(A)** = 1 mm; **(B)** = 500 μm; **(C)** = 100 μm; **(D)** = 20 μm; **(E–G)** = 10 μm.

**Basionym:** *Physarum herbaticum* Shuang L. Chen & Yu Li, *Mycosystema* 19(3):332 (Chen and Li, 2000).

**Description:** Fructifications mainly plasmodiocarps, curved or linear, occasionally producing a single sporocarp, yellow to yellowish-green, sessile. Columella absent. Peridium single-layered, membranous, covered with yellow-green calcareous particles, irregularly dehiscent. Capillitium dense, composed of slender, transparent threads bearing white, polygonal to irregular lime nodes. Spores dark in mass, yellowish-brown by transmitted light, globose, (8.5–) 9–10 μm in diam., minutely warted.

**Habitat:** On decaying leaves.

**Distribution in China:** Guangxi Zhuang Autonomous Region, **Jilin Province**.

**Global distribution:** China.

**Specimens examined:** China, Jilin Province, Dunhua City, Hongye Valley Scenic Area, Hancong Ridge, on decaying leaves, 26 July 2022, X.F. Li, X.Y. Yang (HMJAU M20342).

**Notes:** The transfer of this species to *Nannengaella* is supported by its placement within the *Nannengaella* clade in the multigene phylogeny, together with its concordant morphological features, especially the calcified peridial surface and characteristic lime nodes. Morphologically, *N. herbatica* is distinguished by yellow to yellowish-green plasmodiocarps, a single-layered peridium, white polygonal lime nodes, slender capillitium threads, and the absence of a columella. It resembles *N. lakhanpalii* and *Physarum plicatum* in overall coloration, but differs from *N. lakhanpalii* in lacking fascicled spores and from *P. plicatum* in lacking the wrinkled outer peridium and preformed dehiscence line. It is also comparable to *P. serpula* A.P. Morgan, but that species has larger spores (10–13 μm), whereas *N. herbatica* has smaller spores (8.5–10 μm).

***Nannengaella conglomerata*** ((Fr.) Rostaf.) X.F. Li, B. Zhang **&** Y. Li, comb. nov.

MycoBank: MB855055

Figure 5

**FIGURE 5:**
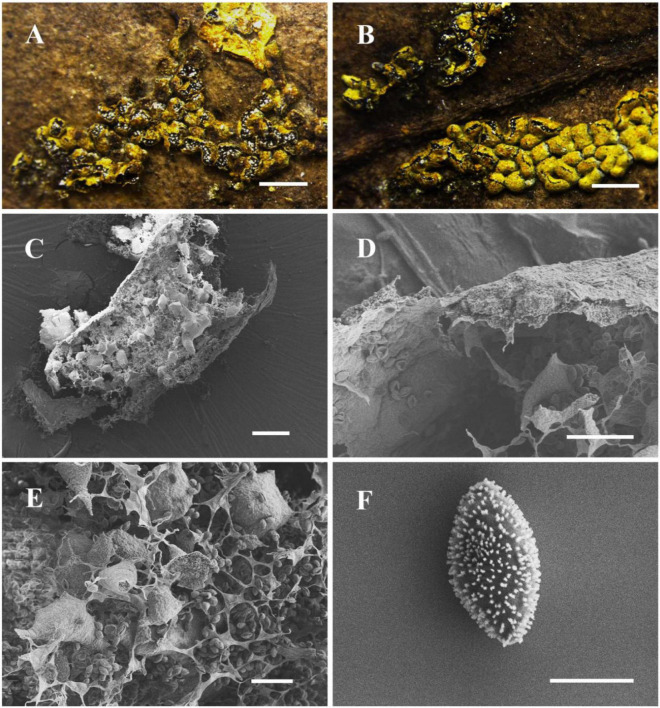
Habitat and microstructure of *Nannengaella conglomerata* (HMJAU M20366). **(A,B)** Sporocarps. **(C)** Plasmodiocarp by SEM. **(D)** Peridium by SEM. **(E)** Capillitium and lime nodes by SEM. **(F)** Spores by SEM. Scale bars: **(A,B)** = 1 mm; **(C)** = 100 μm; **(D,E)** = 40 μm; **(F)** = 5 μm.

**Basionym:** *Diderma conglomeratum* Fr., Syst. mycol. 3(1):111 (1829).

**Description:** Sporocarps or short plasmodiocarps, sessile, densely crowed, often angular due to mutual pressure, pale yellow to yellow, with orange-yellow calcareous particles on the surface. Sporocarps subglobose, 0.3–0.5 mm in diam., plasmodiocarps up to 1 mm. Columella absent. Peridium double-layered, outer layer calcareous, inner layer pale yellow, translucent, membranous, and tightly adherent to the outer layer, dehiscent irregular. Capillitium abundant, transparent, with membranous expansions. Lime nodes white to pale yellow, large, angular or rounded, often aggregated centrally to form a pseudocolumella. Spores dark brown in mass, purple-brown by transmitted light, (8.5–) 9–10 μm in diam., with minutely spinulose.

**Habitat:** On decaying leaves.

**Distribution in China:** Xinjiang Uygur Autonomous Region, Yunnan Province, **Sichuan Province**.

**Global distribution:** China, France, Germany, Spain, the United States, the United Kingdom, Japan, Antigua and Barbuda, the Netherlands, Russia, Ukraine, Finland, India, Romania, Australia.

**Specimens examined:** China, Sichuan Province, Garze Tibetan Autonomous Prefecture, Gexigou National Nature Reserve, on decaying leaves, 14 Aug. 2012, B. Zhang (HMJAU M20366, HMJAU M20332).

**Notes:** The new combination is supported by the placement of this species within the *Nannengaella* clade in the concatenated phylogeny and by its agreement with the morphological circumscription of the genus. *Nannengaella conglomerata* closely resembles *N. contexta* in having pale -yellow plasmodiocarps, a double-layered peridium, white lime nodes, and no columella. However, *N. conglomerata* differs in producing subglobose sporocarps smaller spores ((8.5) 9–10 μm), and frequent pseudocolumella formation. By contrast, *N. contexta* typically forms denser aggregations approaching pseudoaethalia and has larger spores (10–14 μm) ornamented with spinules or warts.

***Nannengaella mellea*** (Berk. & Broome) J.M. García-Martín, J.C. Zamora & Lado, Persoonia 51:110 (García-Martín et al., 2023).

Figure 6

**FIGURE 6:**
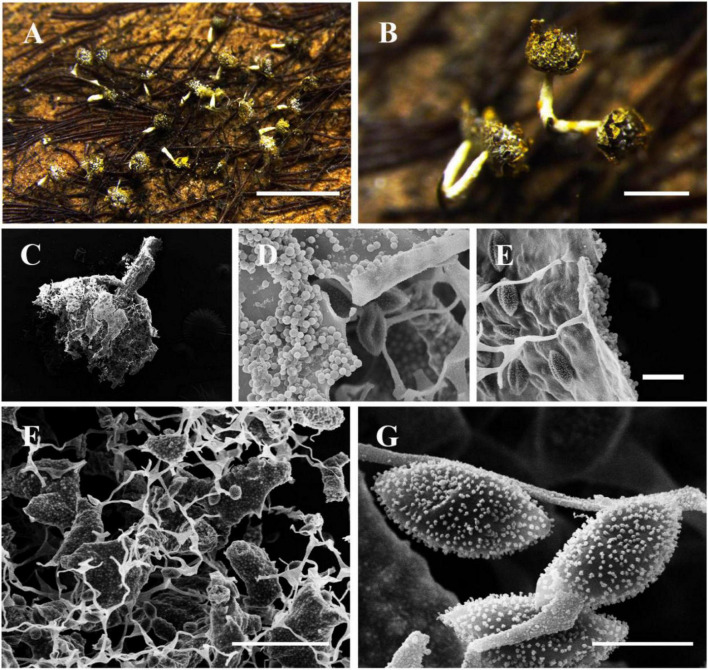
Habitat and microstructure of *Nannengaella mellea* (HMJAU M10204). **(A,B)** Sporocarps. **(C)** Sporocarp by SEM. **(D,E)** Peridium by SEM. **(F)** Capillitium and lime nodes by SEM. **(G)** Spores by SEM. Scale bars: **(A)** = 2 mm; **(B)** = 500 μm; **(E)** = 10 μm; **(F)** = 40 μm; **(G)** = 5 μm.

**Description:** Sporocarps, globose to subglobose, usually stipitate and only rarely sessile, orange-yellow to brownish-orange against an olivaceous-gray background, 0.4–0.6 mm in diam., 0.7–1 mm in total height. Stalk stout, white, calcareous, tapering toward, 0.7–1 mm. Columella present, small, conical, white. Peridium membranous, roughened by a layer of white calcareous particles, dehiscing petaloidally. Hypothallus membranous, transparent. Capillitium reticulate, with large white polygonal lime nodes, 27–95 × 10–40 μm; connecting threads colorless and transparent, with membran ous expansions. Spores dark in mass, light brown by transmitted light, subglobose7–9 (–10) μm in diam., ornamented with clusters of darker warts.

**Habitat:** On decaying leaves.

**Distribution in China:** Beijing City, Hebei Province, Jilin Province, Heilongjiang Province, Jiangsu Province, Anhui Province, Fujian Province, Hubei Province, Hunan Province, Suchuan Province, Yunnan Province, Taiwan Province, Gansu Province, Guangdong Province, Liaoning Province, Shandong Province, Henan Province, Guangxi Zhuang Autonomous Region, Inner Mongolia Autonomous Region, Xizang Autonomous Region, and Hong Kong Special Administrative Region.

**Global distribution:** Widely distributed around the worldwide.

**Specimens examined:** China, Sichuan Province, Liangshan Yi Autonomous Prefecture, Mianning County, Lingshan Temple, on decaying leaves, 12 July 2013, B. Zhang (HMJAU M10106, HMJAU M10295); China, Sichuan Province, Chengdu City, Tazishan Park, on decaying leaves, 15 July 2013, B. Zhang (HMJAU M10158, HMJAU M10206, HMJAU M10100, HMJAU M10108, HMJAU M10166, HMJAU M10104, HMJAU M20875, HMJAU M20876); China, Sichuan Province, Garze Tibetan Autonomous Prefecture, Gexigou National Nature Reserve, on decaying leaves, 14 Aug. 2012, B. Zhang (HMJAU M20863). China, Hubei Province, Suizhou City, Tianhekou Township, Hetao Gou, on decaying leaves, 20 July 2010, B. Zhang (HMJAU M10114, HMJAU M10213). China, Jiangxi Province, Fuzhou City, Junfengshan National Forest Park, on decaying leaves, 19 June 2013, B. Zhang (HMJAU M10171); China, Jiangxi Province, Fuzhou City, Linchuan District, Zhanping Town, on decaying leaves, 9 July 2010, B. Zhang (HMJAU M20880, HMJAU M20881, HMJAU M20882, HMJAU M20883, HMJAU M20884). China, Jilin Province, Changchun City, Jilin Agricultural University Campus, on decaying leaves, 7 Sept. 2015, B. Zhang (HMJAU M20896, HMJAU M20897, HMJAU M20898, HMJAU M20899, HMJAU M20900, HMJAU M20901, HMJAU M20902, HMJAU M20903); China, Jilin Province, Changchun City, Jingyuetan national forest park, on decaying leaves, 1 Aug. 2013, B. Zhang (HMJAU M10169, HMJAU M10165, HMJAU M10139, HMJAU M10175, HMJAU M10172, HMJAU M10116, HMJAU M20871, HMJAU M20872). China, Gansu Province, Tianshui City, Dangchuan Forest Farm, on decaying leaves, 15 Aug. 2010, B. Zhang (HMJAU M20890). China, Yunnan Province, Lijiang City, Zhishan, on decaying leaves, 20 Aug. 2012, B. Zhang (HMJAU M10199, HMJAU M10148, HMJAU M10197, HMJAU M10157, HMJAU M20329). China, Henan Province, Zhumadian City, Biyang County, Tongshan Lake, on decaying leaves, 12 June 2023, B. Zhang (HMJAU M20319, HMJAU M20346, HMJAU M20355). China, Heilongjiang Province, Heihe City, Sunwu County, on decaying leaves, 15 Aug. 2022, X.F. Li (HMJAU M20862, HMJAU M21064, HMJAU M21094). China, Shaanxi Province, Liuba Garden, on decaying leaves, 14 Aug. 2012, B. Zhang (HMJAU M20864). China, Liaoning Province, Fuxin City, Fuxin County, Haitang Mountain Scenic Area, on decaying leaves, 1 Sept. 2012, B. Zhang (HMJAU M20884, HMJAU M20885, HMJAU M20886, HMJAU M20887). China, Guangxi Zhuang Autonomous Region, Baise City, on decaying leaves, 13 July 2017, B. Zhang (HMJAU M20894). China, Shandong Province, on decaying leaves, 24 Jan. 1905, Y. Li (HMJAU M20895). China, Jiangsu Province, Nanjing City, Nanjing Agricultural University, on decaying leaves, 8 June 2016, B. Zhang (HMJAU M20913).

**Notes:** *Nannengaella mellea* is a common and variable species, especially in sporocarp coloration, which ranges from honey yellow and orange-yellow to grayish-yellow. It may be confusion with *Physarum citrinum*, owing to because of the similar sporocarp color and small columella. However, *N. mellea* differs in having polygonal lime nodes and a shorter, stouter stalks, whereas *P. citrinum* typically has rounded lime nodes and a more slenderer stalks.

***Nannengaella sulphurea*** (Alb. & Schwein.) J.M. García-Martín, J.C. Zamora & Lado, *Persoonia* 51:110 (García-Martín et al., 2023).

Figure 7

**FIGURE 7:**
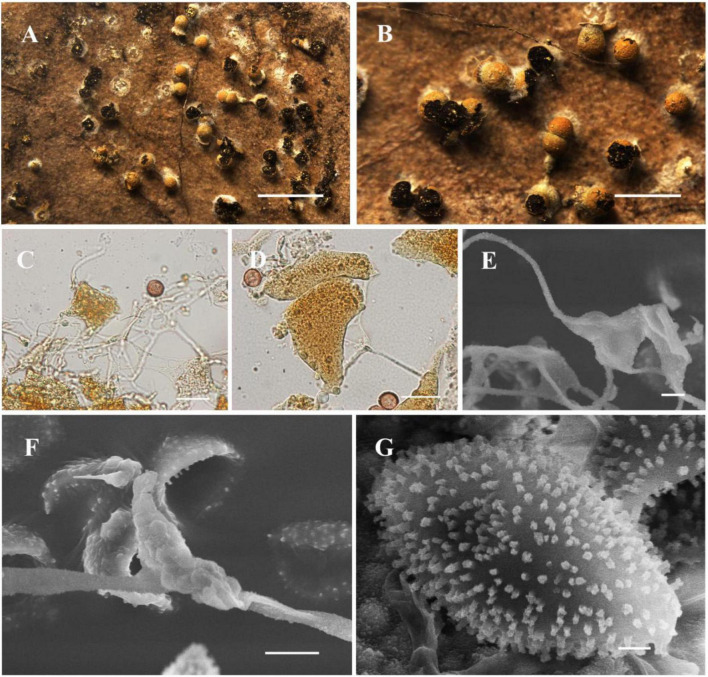
Habitat and microstructure of *Nannengaella sulphurea* (HMJAU M20341). **(A,B)** Sporocarps. **(C–F)** Capillitium and lime nodes by TL and SEM. **(G)** Spores by SEM. Scale bars: **(A)** = 2 mm; **(B)** = 1 mm; **(C,D)** = 20 μm; **(E)** = 2 μm; **(F)** = 4 μm; **(G)** = 1 μm.

**Description:** Sporocarps gregarious, stipitate, cylindrical to clavate, pale yellow to pale ochraceous. Stalk short, calcareous, usually thicker at the base, white or yellowish brown. Columella present as a white conical protrusion. Hyporhallus white, calcareous. Peridium membranous, semi-transparent, covered with orange-yellow calcareous scales, irregularly dehiscent. Capillitium reticulate, colorless and transparent, with membranous and elongated expansions. Lime nodes large, angular, yellowish to white, sometimes forming a pseudocolumella. Spores dark in mass, crineous under cinereous by transmitted light, 9–10 (–11) μm in diam., warted, with warts occasionally arranged in lines.

**Habitat:** On decaying leaves.

**Distribution in China:** Beijing City, Hebei Province, Hubei Province, Anhui Province, Hunan Province, **Sichuan Province.**

**Global distribution:** China, the United States, Canada, India, Mexico, Norway, Brazil, Japan, Germany, Russia, Argentina, Puerto Rico, Sierra Leone.

**Specimens examined:** China, Sichuan Province, Garze Tibetan Autonomous Prefecture, Gexigou National Nature Reserve, on decaying leaves, 3 Aug. 2014, B. Zhang (HMJAU M20333, HMJAU M20334, HMJAU M20341); China, Sichuan Province, Garze Tibetan Autonomous Prefecture, Gexigou National Nature Reserve, on decaying leaves, 14 Aug. 2012, B. Zhang (HMJAU M21278, HMJAU M21279, HMJAU M20288).

**Notes:** This species is readily recognized by its pale yellow to ochraceous, cylindrical to clavate sporocarps, and a peridium roughened with orange-yellow calcareous scales, and distinct conical columella. It resembles *Physarum auriscalpium* in sporocarp color and spore size, but differs in its more robust calcareous structures and in the presence of a conical columella.

***Nannengaella leucopus*** (Link) J.M. García-Martín, J.C. Zamora & Lado, *Persoonia* 51:110 (García-Martín et al., 2023).

Figure 8

**FIGURE 8:**
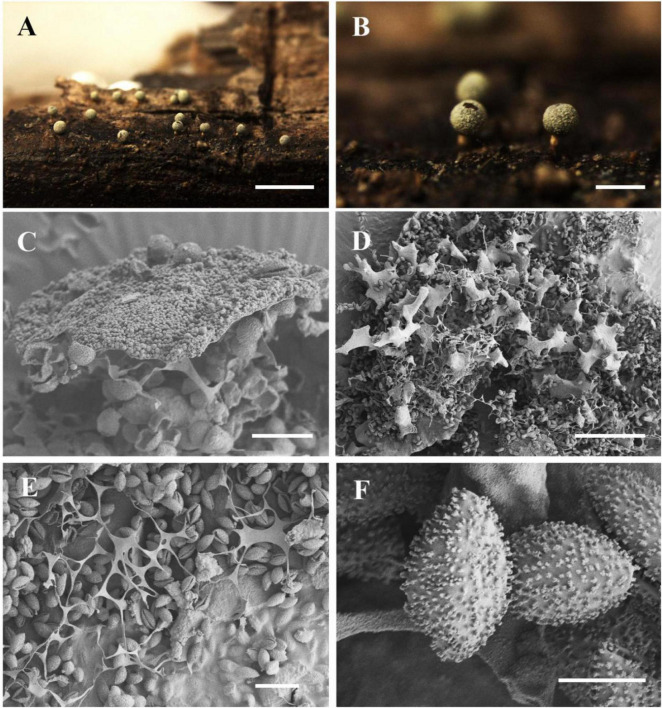
Habitat and microstructure of *Nannengaella leucopus* (HMJAU M20301). **(A,B)** Sporocarps. **(C)** Peridium by SEM. **(D,E)** Capillitium and lime nodes by SEM. **(F)** Spores by SEM. Scale bars: **(A)** = 2 mm; **(B)** = 500 μm; **(C,E)** = 20 μm; **(D)** = 100 μm; **(F)** = 5 μm.

**Description:** Sporocarps gregarious, erect, short-stalk or sessile, light yellow, rough, subglobose, sometimes slightly umbilicate below, 0.3–0.4 mm in diam., and about 0.5 mm in total height. Stalk thick, cylindric, slightly tapering upwards, white to yellowish-white, calcareous, 0.2–0.25 mm long. Columella present, small, sometimes expressed as a short conical protuberance, yellowish-white. Hypothallus membranous, transparent, yellow-brown to light yellow. Peridium single-layered, membranous, roughened with white granular scales, light yellow, irregularly dehiscent, lacking calcium at the junction with the stalk and often showing a iridescence. Capillitium loosely reticulate, colorless and transparent, with large light-yellow to white lime nodes, that are elongate to angular, sometimes forming an irregular pseudocolumella. Spores dark in mass, light brown by transmitted light, 9–10 (–11) μm in diam., warted.

**Habitat:** On decaying woods.

**Distribution in China:** Beijing City, Hebei Province, Jilin Province, Jiangsu Province, Zhejiang Province, Anhui Province, Fujian Province, Yunnan Province, Taiwan Province, Heilongjiang Province, Inner Mongolia Autonomous Region, Hainan Province, Gansu Province, Guangdong Province, Liaoning Province, **Sichuan Province**.

**Global distribution:** China, the United States, Germany, Mexico, Russia, the Netherlands, France, Canada, Spain, Argentina, and the United Kingdom.

**Specimens examined:** China, Sichuan Province, Garze Tibetan Autonomous Prefecture, Gexigou National Nature Reserve, on decaying woods, 14 Aug. 2012, B. Zhang (HMJAU M20301). China, Heilongjiang Province, Heihe city, Sun Wu County, near Yijia Mountain, on decaying woods, 15 Aug. 2022, X.F. Li (HMJAU M21058, HMJAU M20364, HMJAU M21088, HMJAU M20327). China, Inner Mongolia Autonomous Region, Motianling, on decaying woods, 15. Aug. 1985, Y. Li, S.L. Chen (HMJAU 9123).

**Notes:** *Nannengaella leucopus* is a widespread species characterized by its short, robust calcareous stalk, pale-yellow sporocarps, and larger spores relatively [9–10 (–11) μm]. It is morphologically similar to *N. globulifera* and *Physarum citrinum*. However, *N. globulifera* has smaller spores, whereas *P. citrinum* differs in having smaller, rounded lime nodes and a golden stalk, rather than the elongate to angular lime nodes and white to yellowish-white stalk typical of *N. leucopus*.

***Nannengaella contexta*** (Pers.) J.M. García-Martín, J.C. Zamora & Lado, *Persoonia* 51:110 (García-Martín et al., 2023).

Figure 9

**FIGURE 9:**
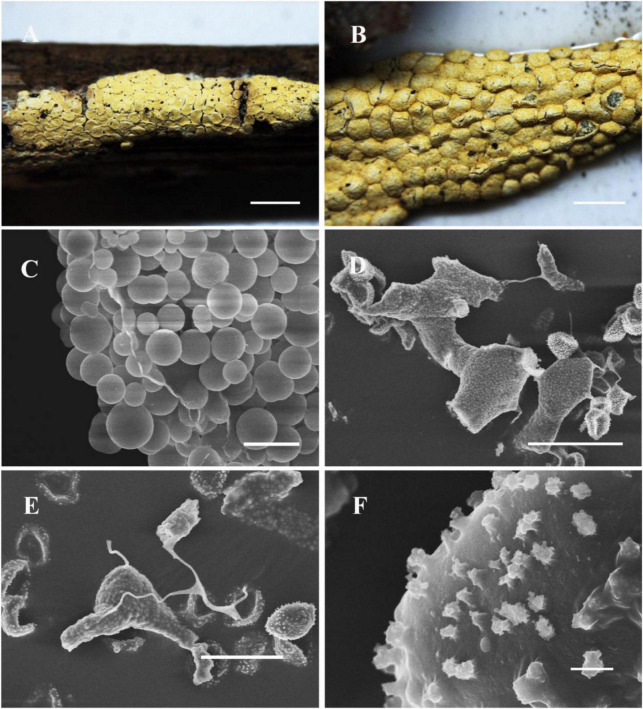
Habitat and microstructure of *Nannengaella contexta* (HMJAU M20354). **(A,B)** Sporocarps. **(C)** Peridium by SEM. **(D,E)** Capillitium and lime nodes by SEM. **(F)** Spores by SEM. Scale bars: **(A,B)** = 1 mm; **(C)** = 2 μm, **(D)** = 40 μm, **(E)** = 20 μm, **(F)** = 1 μm.

**Description:** Sporocarps or short plasmodiocarps, gregarious, pale yellow to yellow, densely grouped and often angular due to mutual pressure, but not superimposed, ovoid to reniform, sessile. Columella absent. Peridium double-layered, outer layer thick, calcareous, pale yellow, inner layer membranous, pale to yellowish. Capillitium dense, with white to yellowish, angular lime nodes, sometimes confluent in the center to form a pseudocolumella. Spores dark in mass, reddish-brown by transmitted light, 10–11.0 (–11.5) μm in diam., with densely warted to spinulose.

**Habitat:** On decaying leaves.

**Distribution in China:** Heilongjiang Province, Jilin Province, Henan Province, Gansu Province, Shandong Province, Yunnan Province, Xizang Autonomous Region, Inner Mongolia Autonomous Region, Sichuan Province.

**Global distribution:** China, the United States, Canada, France, Germany, the United Kingdom, the Netherlands, Spain, Russia, Mexico, Sweden, India, Japan, Pakistan.

**Specimens examined:** China, Sichuan Province, Garze Tibetan Autonomous Prefecture, Gexigou National Nature Reserve, on decaying leaves, 3 Aug. 2014, B. Zhang (HMJAU M20354); China, Sichuan Province, Garze Tibetan Autonomous Prefecture, Gexigou National Nature Reserve, on decaying leaves, 14 Aug. 2014, B. Zhang (HMJAU M21297, HMJAU M20340, HMJAU M20289, HMJAU M20339).

**Notes:** *Nannengaella contexta* is distinguished by pale-yellow sporocarps that become angular by mutual pressure, the absence of a columella, double-layered peridium, and angular lime nodes. It resembles *Physarum conglomeratum* in having yellow sporocarps and a two-layered peridium, but differs in its flatter to concave sporocarps with angular margins, larger spores (10–11.0 (–11.5) μm), and more regular dehiscence. By contrast, *P. conglomeratum* has round to convex plasmodiocarps, irregular dehiscence, and smaller spores (8–10 μm). *Physarum tessellatum* differs further in having white sporocarps, rounded lime nodes, and a distinctly tessellated calcareous peridium.

***Nannengaella plicata*** (Nann. - Bremek. & Y. Yamam.) J.M. García-Martín, J.C. Zamora & Lado, *Persoonia* 51:110 (García-Martín et al., 2023).

Figure 10

**FIGURE 10:**
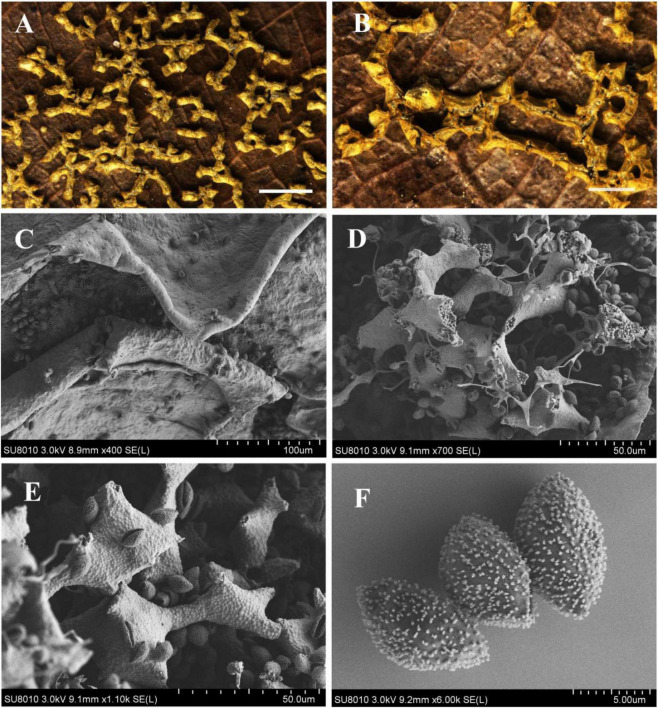
Habitat and microstructure of *Nannengaella plicata* (HMJAU M20348). **(A,B)** Plasmodiocarps. **(C)** Peridium by SEM. **(D,E)** Capillitium and lime nodes by SEM. **(F)** Spores by SEM. Scale bars: **(A)** = 2 mm; **(B)** = 1 mm.

**Description:** Fructifications mainly plasmodiocarps, scattered to gregarious, reticulate or branched, cylindric, curved, and distinctly wrinkled; yellow, orange-yellow or greenish yellow, sessile. Columella absent. Peridium double-layered; both layer membranous, light yellow, with white calcareous particles deposited between them, dehiscence occurring along a preformed longitudinal line, irregular, the remaining areas smooth. Hypothallus membranous, transparent, yellowish. Capillitium dense, reticulate, with numerous small and white angular lime nodes, 25–65 × 15–40μm. Spores dark brown in mass, light brown by transmitted light, subglobose, (8.5–) 9–10 μm in diam., warted.

**Habitat:** On decaying leaves.

**Distribution in China:** Taiwan Province, Henan Province, Jilin Province, **Sichuan Province.**

**Global distribution:** China, Japan, Democratic Republic of Congo, Equatorial Guinea, Nepal.

**Specimens examined:** China, Henan Province, Zhumadian City, Biyang County, Wanfeng Temple, on decaying leaves, 12 June 2023, B. Zhang (HMJAU M20348, HMJAU M21290, HMJAU M20371, HMJAU M21102, HMJAU M21103); China, Henan Province, Zhumadian City, Biyang County, Tongshan Lake, on decaying leaves, 12 June 2023, B. Zhang (HMJAU M21101); China, Henan Province, Zhumadian City, Biyang County, botanical garden, on decaying leaves, 11 June 2023, B. Zhang (HMJAU M20320, HMJAU M20321, HMJAU M21124, HMJAU M21125, HMJAU M21126, HMJAU M21127, HMJAU M21128, HMJAU M21129, HMJAU M21130, HMJAU M21131, HMJAU M21132, HMJAU M21133, HMJAU M21134). China, Sichuan Province, Garze Tibetan Autonomous Prefecture, Gexigou Nature Reserve, on decaying leaves, 14 Aug. 2012, B. Zhang (HMJAU M20303).

**Notes:** This species is characterized by its yellow plasmodiocarps with distinct longitudinal folds, a double-layered peridium with a preformed dehiscence line, and numerous small white angular lime nodes. It resembles *Physarum aeneum* in having yellow plasmodiocarps and a two-layered peridium, but differs in its larger spores ((8.5–) 9–10 μm vs. 7–9 μm), white angular lime nodes rather than pale yellow rounded ones, and a membranous rather than cartilaginous outer peridium.

**Key to the species of *Nannengaella***

Note: This key includes all currently recognized species of *Nannengaella*, including taxa not treated in detail in the present study (*N. alpestris*, *N. alpina*, *N. globulifera*, *N. lakhanpalii*, and *N. laevis*).

1. Fructifications mainly plasmodiocarps or aethalia…………2

1. Fructifications mainly sporocarps…………°……………3

2. Fructification forming aethalia.……………..*Nannengaella laevis*

2. Fructification forming plasmodiocarps.…………………..4

3. Columella present……………………………………5

3. Columella absent……………………………………..6

4. Plasmodiocarps lacking a preformed dehiscence line……….…….°……………7

4. Plasmodiocarps with preformed dehiscence line, producing longitudinal irregular folds.……………………………………8

5. Sporocarps pale yellow to pale ochraceous, cylindrical or clavate.….……………………………..*Nannengaella sulphurea*

5. Sporocarps rounded, not as above ……………….…….………….…….9

6. Sporocarps cream to lemon yellow, stipitate……………….*Nannengaella cremilutea*

6. Sporocarps yellow to pale yellow, sessile……………………….….….………0.10

7. Peridium double-layered; sporocarps dark yellow.……0.11

7. Peridium single-layered; sporocarps yellow to yellowish-green……………………………*Nannengaella herbaticum*

8. Lime nodes white, polygonal…….…. *Nannengaella plicata*

8. Lime nodes yellow, circular to fusiform.*Nannengaella lakhanpalii*

9. Stalk 0.6–1.0 mm long; spores < 9 μm……….….…12

9. Stalk 0.2–0.25 mm long; spores > 9 μm…….….…….…*Nannengaella leucopus*

10. Sporocarps with orange-yellow surface scales, spores 9–10 μm.……………….…………….…… *Nannengaella conglomeratum*

10. Sporocarps without orange-yellow surface scales; spores 10–14 μm.…13

11. Spores <10 μm.*Nannengaella luteotestacea*

11. Spores >10 μm.*Nannengaella alpestris*

12. Sporocarps light yellow, honey yellow, or dark yellow.*Nannengaella mellea*

12. Sporocarps white.*Nannengaella globulifera*

13. Lime nodes white or yellowish.*Nannengaella contexta*

13. Lime nodes yellow or light yellow*.Nannengaella alpina*

## Discussion

This study refines the taxonomy of *Nannengaella*, a recently established genus within *Physaraceae*, through an integrative framework combining multilocus phylogenetics and detailed morphological analyses of specimens collected across a broad geographic range in China. Our analyses consistently recovered *Nannengaella* as a distinct and well-supported lineage separate from *Physarum* and related genera, thereby supporting the generic circumscription proposed by García-Martín et al. (2023). By incorporating five molecular markers (nSSU, EF-1α, mtSSU, α-Tub, COI), the present study provides a robust basis for evaluating taxa in a group where traditional classification has long been complicated by overlapping morphological characters, particularly sporocarp architecture, peridial structure, and lime deposition patterns (Nandipati et al., 2012; Chen and Li, 2000; Ronikier et al., 2022; García-Martín et al., 2023; Prikhodko et al., 2023). These findings further highlight the central role of molecular phylogenetics in resolving taxonomic complexity in morphologically variable myxomycete groups.

The recognition of *Nannengaella luteotestacea* expands the currently known diversity of the genus. In the concatenated phylogeny, this taxon formed a distinct lineage (Figure 1), and its morphology was likewise diagnostic, especially the dark-yellow plasmodiocarps, double-layered peridium, absence of a preformed dehiscence line, and small warted spores (Figure 2). Its distinction from morphologically similar taxa such as *Physarum serpula* and *N. plicata* illustrates the value of combining morphology with multilocus evidence in species delimitation. This integrative approach is particularly important in myxomycetes, where morphological convergence and intraspecific variation can obscure taxonomic boundaries and potentially conceal cryptic diversity.

Our results also support the transfer of three previously described *Physarum* species, *N. herbatica*, *N. cremilutea*, and *N. conglomerate* to *Nannengaella* (Tice et al., 2016; Liu and Chen, 1998). These recombinations are supported not only bytheir phylogenetic placement within the *Nannengaella* clade, but also by morphological features consistent with the genus, including calcified fructifications, characteristic lime nodes, and, in some taxa, pseudocolumella formation. Together, these changes strengthen the ongoing revision of *Physaraceae* initiated by García-Martín et al. (2023) and contribute to a more stable delimitation of *Nannengaella*. Rather than merely increasing the number of recognized taxa, these findings help reduce the taxonomic ambiguity historically associated with the broad and heterogeneous concept of *Physarum*.

The present study also expands the known distribution of *Nannengaella* in China. We documented the first report of *N. herbatica* from Jilin Province, significant substantial range extensions for *N. cremilutea* into Heilongjiang, Shaanxi, and Henan Provinces, and new records of *N. conglomerata*, *N. contexta*, *N. sulphurea*, and *N. leucopus* from Sichuan Province. These findings indicate that some species are more widely distributed than previously recognized especially *N. cremilutea* and *N. mellea*. At the same time, the current distributional pattern likely still reflects uneven sampling intensity rather than the full extent of species ranges. Myxomycete species exhibit different substrate selectivity, but most prefer decaying wood and litter (Lin et al., 2024). Most collections were made in forested habitats rich in decaying plant material, such as most specimens collected from Sichuan’s Gexigou Reserve and Jilin’s Changbai Mountains in this study. Nevertheless, ecological interpretation remains limited because environmental variables such as substrate type, humidity, pH, and microclimatic conditions were not systematically quantified (Schnittler and Stephenson, 2000; Schnittler, 2001; Stephenson et al., 2003; Lado et al., 2007; Novozhilov and Schnittler, 2008; Tice et al., 2016; Dagamac et al., 2017). Future studies integrating taxonomic with habitat metadata will be important for understanding ecological preferences, local persistence, and range limits within the genus.

Although the overall phylogenetic framework was well resolved, some internal relationships within *Nannengaella* received only moderately support. This may reflect recent divergence, incomplete lineage sorting, insufficient resolution of the currently sampled loci. Limited taxon coverage for some markers may also have contributed to reduced support at deeper or intermediate nodes. These results suggest that, while the present multilocus dataset is sufficient for delimiting major lineages and supporting several taxonomic decisions, additional data will be needed to clarify finer-scale relationships within the genus. Expanding genomic sampling, including phylogenomic approaches or broader locus representation, may help improve resolution and reveal overlooked diversity (Leontyev et al., 2019; Shchepin et al., 2019), particularly in morphologically variable taxa such as *N. mellea* (Figure 6), in which color variation may obscure underlying lineage structure.

The detailed taxonomic treatments provided here, including SEM observations of peridium, capillitium, and spore ornamentation (Figures 2–10), also highlights the continuing importance of morphology in myxomycete systematics. Features such as spore ornamentation, lime-node shape, and peridial architecture proved especially informative when interpreted in combination with phylogenetic evidence (Nandipati et al., 2012; Leontyev et al., 2019; García-Martín et al., 2023). In this study, these characters were critical for distinguishing closely related taxa and for diagnosing the new species and new combinations. At the same time, the taxonomic utility of some structures remains incompletely understood because their developmental variation has rarely been studied in detail. Further work on ontogenetic changes in lime deposition, peridium differentiation, and capillitium development could improve character interpretation and provide additional insight into evolutionary trends within *Physaraceae* (Everhart, 2008).

In summary, this study provides an expanded and better-resolved taxonomic framework for *Nannengaella* by integrating multilocus phylogenetic evidence, comparative morphology, and new distributional data from China. The recognition of a new species, the proposal of three new combinations, and the documentation of multiple range extensions collectively improve current understanding of the genus. Although ecological and developmental questions remain open, the present results provide a stronger basis for future studies on the diversification, biogeography, and evolutionary ecology of *Nannengaella* and related physaraceous myxomycetes.

## Data Availability

The datasets presented in this study can be found in online repositories. The names of the repository/repositories and accession number(s) can be found in the article/Supplementary material.
